# The PROMISE study: protocol for a community-based cohort study and co-creation of a reproductive health intervention in Somaliland

**DOI:** 10.1080/16549716.2026.2685408

**Published:** 2026-06-22

**Authors:** Monica Lauridsen Kujabi, Farduus Mohamed, Mohamed Mussa Abdilahi, Nanna Maaløe, Gileard Masenga, Annemette Wildfang Lykkebo, Jonah Kiruja, Vibeke Rasch, Soheir Hassan Ahmed

**Affiliations:** a Department of Public Health, University of Copenhagen, Copenhagen, Denmark; b Department of Obstetrics and Gynaecology, Randers Regional Hospital, Randers, Denmark; c School of Graduate Studies, University of Hargeisa, Hargeisa, Somalia; d College of Medicine and Health Sciences, University of Hargeisa, Hargeisa, Somaliland; e Department of Obstetrics and Gynaecology, Copenhagen University Hospital - Hvidovre, Hvidovre, Denmark; f Department of Public Health and Health Systems, Kilimanjaro Christian Medical College University, Tanzania; g Department of Obstetrics and Gynaecology, Odense University Hospital, Odense, Denmark; h Department of Clinical Research, University of Southern Denmark, Odense, Denmark

**Keywords:** antenatal care, maternal near miss, social support, collective decision-making, co-creation

## Abstract

Somaliland faces persistently high burdens of maternal and perinatal mortality, with limited population-based data on pregnancy complications, sociocultural influence on maternal and perinatal health, and women’s reproductive health needs across the continuum of pregnancy, childbirth, and postpartum. Also, available health materials, such as for assessing maternal near misses (MNM) or strengthening healthcare literacy, often appear unfit in the contextual realities. In response, the objective of this study is to unfold the physical, cultural, and psychosocial strengths and challenges experienced by women in Somaliland during pregnancy, childbirth, and the postpartum period; to examine how these factors as well as the woman’s health-seeking behaviour influence pregnancy outcomes and women’s ability to achieve future reproductive health goals; and to pilot how these insights can inform the co-creation of context-appropriate health materials. The PROMISE study is a community-based longitudinal pregnancy cohort in Hargeisa, Somaliland, including approximately 800 pregnant women <28 weeks of gestation recruited from randomly selected sub-districts. Women will be followed up at three time points (<28 weeks of gestation, >36 and one-three months postpartum) using questionnaires and clinical measurements. An MNM tool will be adapted through a Delphi process, and its validity will be tested using the cohort. The cohort findings will inform a co-creation process to develop postpartum contraceptive counselling materials to be pilot-tested for feasibility, acceptability, and preliminary effects. This protocol responds to major evidence gaps in fragile and low-resource settings, and aims to generate contextually grounded evidence and co-created interventions to strengthen maternal health agency in Somaliland and beyond.

## Paper Context

- **Main findings:** This study will generate the first longitudinal, community-based pregnancy cohort in Somaliland capturing the physical, mental, and sociocultural determinants of maternal health across pregnancy, birth, and postpartum periods.
- **Added knowledge:** By embedding multiple sub-studies within a single community-based pregnancy cohort, this study will link clinical and epidemiological findings to sociocultural determinants, such as social support, self-care, and collective decision-making.
- **Global health impact for policy and action:** Findings can inform context-sensitive policies and co-created interventions, including postpartum contraception counselling, to strengthen reproductive health services and agency.

## Background

In 2023, an estimated 260,000 women died globally due to pregnancy-related complications, and approximately five million babies were stillborn or died within the first month of life [1,2]. Sub-Saharan Africa bears a disproportionate share of this global burden, accounting for nearly 70% of all maternal and neonatal deaths [2]. While Somaliland has made progress in improving maternal and newborn health, it remains among the countries with the highest maternal and perinatal mortality ratios, reporting 396 maternal deaths per 100,000 live births and 36 stillbirths per 1,000 total births [2,3]. Most of these deaths are preventable with timely and adequate care [2]. Moreover, it is expected that many women and babies survive with physical and mental consequences, with long-lasting effects on their lives, their resistance to external shocks such as natural disasters, conflicts, and disease outbreaks, and impacting the lives of their families and communities [4].

The factors contributing to poor outcomes of pregnancy and childbirth are complex and multifactorial. A recent study from Somaliland found that 78% of maternal deaths occurred upon arrival at health facilities, highlighting delayed care-seeking, infrastructural, and systemic barriers [5]. Structural barriers such as out-of-pocket costs, lack of transportation, shortages of skilled health providers, and limited medical supplies compounded by sociocultural factors and health system weaknesses further exacerbate the situation [4,6]. The low uptake of health services is evident in the Somaliland Health and Demographic Survey, which reports antenatal care (ANC) coverage at 47% and skilled birth attendance at 33% [4]. Among the less than half women attending at least one ANC visit, a recent prospective cohort study found that fewer than half of them attended four or more visits, and inadequate ANC attendance was associated with antepartum haemorrhage, caesarean section, preterm birth, neonatal intensive care unit admission, and stillbirth compared with women attending at least four visits [7]. In particular, multiparous women in Somaliland experience disrespectful and poor-quality care, discouraging them from seeking facility-based care [8].

Strengthening ANC utilization is critical for the early identification of complications, promoting skilled birth attendance, ensuring that women’s individual reproductive health goals are met and that health information is conveyed with a life course and inter-generational perspective [9]. ANC is the most common routine medical contact for women and offers an, often underutilized, opportunity to provide health guidance within and beyond their current pregnancy [10,11]. Pregnancy complications, such as obesity, hypertensive disorders, gestational diabetes, and anemia, are well-established predictors of life course and intergenerational health risks [10]. Providing women with this information, along with contextualized lifestyle guidance for secondary prevention, is crucial, but rarely implemented [10].

Against this background, understanding women’s reproductive health in Somaliland and how they navigate their pregnancy, is essential. This is the overarching focus of this research project. We further explore four embedded topics in greater depth.

First, gender imbalances significantly influence maternal outcomes [12,13]. For example, 98% of women have undergone female genital mutilation, which causes long-term physical and psychological effects [12]. Traditional patriarchal norms and collective decision-making processes constrain women’s health agency [13]. Particularly in emergencies, the need for consent from a male relative before medical care can delay access to life-saving care [14,15]. Reproductive health is also shaped by psychosocial, cultural, and relational factors that influence women’s experiences during pregnancy and the postpartum period. Previous research from low- and middle-income countries has highlighted the importance of social support, decision-making dynamics, mental health, gender norms, and reproductive agency in shaping care-seeking behaviour, contraceptive use, and maternal wellbeing [13–17]. In Somaliland, social support and collective decision-making processes appear central to understanding care-seeking behavior and delays [6]. Little is known about how these community and family dynamics interact with socioeconomic constraints, gender relations, and health system weaknesses to prolong care delays [18,19].

Second, Somaliland faces a rising burden of non-communicable diseases, including obesity, hypertension, and diabetes [20,21]. Globally, gestational diabetes affects approximately one in six pregnancies, while hypertensive disorders affect 2–15%, with higher prevalence in low-income settings [22,23]. These conditions each, and when co-occurring, markedly elevate the risk of adverse outcomes such as cesarean delivery, preterm birth, and long-term chronic illness for both mother and baby. Although data on gestational diabetes and hypertensive disorders in pregnancy in Somaliland are scarce, growing trends in obesity and diabetes, together with limited ANC coverage, suggest a growing risk. Many affected women remain undiagnosed or receive delayed treatment owing to inadequate screening and care infrastructure [20]. Notably, among young women in Somaliland, 37% have essential hypertension, which markedly increases the risk of superimposed pre-eclampsia [20].

Third, in Somaliland, modern contraceptive use remains exceptionally low at 1% of women of reproductive age [4]. It is estimated that if the unmet need for family planning in sub-Saharan Africa is met, it can potentially reduce the number of unintended pregnancies and unsafe abortions by up to 78% [24]. Importantly, unsafe abortions contribute significantly to maternal mortality. Postpartum family planning represents a critical yet underutilized opportunity [25,26]. Cultural and religious beliefs, fear of side effects, inadequate counselling, myths, and the need for male consent are among the key barriers to uptake [14,26–28].

Finally, the challenges of maternal and perinatal short- and long-term adverse events, as well as suboptimal health agency and reproductive rights, may cause profound risks of mental ill health. Compounding these challenges is the often-overlooked ‘epidemic of grief’ arising from the global burden of preventable stillbirths and neonatal deaths [29]. Many newborns who survive face chronic health conditions, further intensifying the emotional and social toll on mothers and families. Despite these aspects, no studies have estimated the burden of perinatal depression in Somaliland.

These health crises not only impact women’s immediate wellbeing but also have long-lasting effects on their lives, their resistance to external shocks such as natural disasters, conflicts, and disease outbreaks, and the lives of their families and communities. Addressing the complex challenges related to maternal health in Somaliland requires a holistic approach that integrates medical, psychosocial, cultural and structural dimensions of maternal health. Social support, perceived quality of care, gender norms, and women’s lived experiences shape the entire reproductive healthcare continuum.

Against this background, the PROMISE (Promoting Reproductive Health in Somaliland for Empowerment) study presents a community-based prospective longitudinal cohort study. This study seeks to generate contextually grounded evidence to inform targeted interventions and health policies in Somaliland and beyond. It is of paramount importance that such rich data lead to action. Therefore, an embedded intervention is planned applying postpartum contraceptives as a case for how the cohort study’s findings can generate change through a co-creation process.

### Overall objective

To unfold the physical, cultural, and psychosocial strengths and challenges experienced by women in Somaliland during pregnancy, childbirth, and the postpartum period; to examine how these factors interact to influence pregnancy outcomes and women’s ability to achieve future reproductive health goals; and to pilot how these insights can inform the co-creation of context-appropriate health interventions.

### Specific objectives

1. To adapt and validate the sub-Saharan Africa maternal near miss (MNM) tool, originally created for health facility settings, for future use in community settings, to further quantify the burden of severe maternal outcomes (SMOs), also outside the healthcare system, and their association with collective decision-making.
2. To investigate whether number of ANC visits and facility births are associated with a composite of adverse pregnancy outcomes as well as whether women’s levels of social support and self-care are associated with healthcare service uptake and meeting future reproductive health goals. Further, to investigate women’s pregnancy journeys, including their contraceptive knowledge, attitudes, practices, and physical and mental health.
3. Based on the cohort findings, to co-create and pilot-test information materials aimed at improving uptake of postpartum contraception and supporting women in meeting their reproductive health goals.
4. To conduct a secondary analysis, triangulating the findings from all studies to gain a comprehensive understanding of strengths and challenges in maternal and perinatal health in Somaliland informing policy and action.

## Methods

### Study organization

This study is based on a collaboration between University of Hargeisa, Somaliland, Kilimanjaro Christian Medical College University, Tanzania, University of Southern Denmark, and University of Copenhagen, Denmark. The study is part of the Building Stronger Universities phase IV collaboration, which is funded by the Danida Fellowship Centre. The study team includes two PhD students, three postdoctoral researchers, five senior researchers, seven master’s students and sixteen trained research assistants with a background in nursing/midwifery. The researchers’ backgrounds cover clinical obstetrics, social sciences, implementation science, epidemiology, and statistics.

### Study setting

Somaliland is a semi-desert, partially recognized independent country located in the Horn of Africa, bordered by Djibouti, Ethiopia, and Somalia [4]. Spanning an area of 176,119 square kilometers with 850 kilometers of coastline along the Gulf of Aden, it declared its independence from Somalia in 1991 [4]. At present, the population is projected to be 4.8 million (based on the Population Estimation Survey from 2014, growth rate 2.9%) while a government estimate states 6.2 million [30]. Somaliland comprises five regions. The study will be conducted in Hargeisa, the capital city of Somaliland, and one of the largest districts in the Maroodi Jeeh region. Hargeisa has experienced rapid growth from 860,000 people in 2012 to 1.5 million in 2020. Administratively, Hargeisa is divided into eight districts: Mohamed Mooge, Ahmed Dhagah, Gacanlibaax, 26 June, Ahmed Ma’alin Haaruun, 31 May, Mahmoud Haibe, and Ibrahim Kodbuur.

Due to conflicts and recurrent droughts, formal and informal settlements have evolved over time. Internally displaced persons constitute approximately 10%, a number predicted to increase as climate change increasingly takes its toll over the country [30]. In contrast, the nomadic population has decreased due to urbanization, drought, and loss of livestock, and now accounts for 34% of the population [31]. The main sources of income include wage employment, agriculture, livestock, and remittances from the diaspora. In Somaliland, 41% of households have access to improved drinking water, life expectancy is 52 years, 48% of the population is under the age of 15, and the fertility rate is 5.6 [4].

Gender inequalities are profound in Somaliland, with 41% of the women being illiterate. Of females, 26% have never attended school, while the number is 17% for males. According to the demographic health survey, physical abuse occurred in 12% females, 9% females were married before the age of 15, and 23% before the age of 18 years [4]

The Somaliland health system is primarily financed through out-of-pocket expenditure. There are approximately 355 health facilities in Somaliland, and approximately 50% of these facilities are located in the capital region, Maroodi Jeeh [32]. The health system has a pyramidal structure with primary health units, maternal and child health units (MCH), district hospitals, and referral hospitals where specialized care takes place [30]. Due to a massive human resources for health crisis, only hospitals are staffed by doctors and have access to cesarean sections and blood transfusions. There are two government hospitals in Hargeisa City and several private hospitals [30].

Somaliland’s community structure is deeply rooted in clan-based and extended family networks, which influences health-seeking behaviors [33]. Decision-making authority commonly rests with male household heads and clan elders, limiting women’s autonomy in accessing maternal health services [8]. While women play an essential role in household and community life, their maternal health choices are often shaped by collective family and clan dynamics rather than by individual agency. Maternal healthcare decisions are negotiated within broader social frameworks [34]. Traditional birth attendants (local uneducated birth companions) remain trusted maternal care providers [33,35].

### Study design

**Figure 1.**
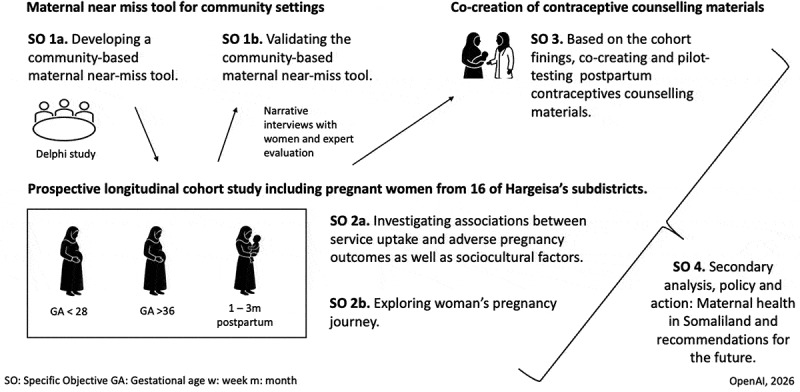
PROMISE study design.

An interdisciplinary approach relying on mixed-methods research will be utilized. The primary study will be a prospective, longitudinal cohort study. Nested within this study are adaptation of a community-based MNM tool and co-creation of contraceptive counselling materials. The components are shown in Figure 1 and are described in the following. Qualitative components include interviews with MNM cases and interviews related to the contraceptive counselling intervention. The study will be carried out in accordance with the principles of the Declaration of Helsinki [36].

The PROMISE study centers around a longitudinal prospective cohort study of around 800 pregnant women in Hargeisa, Somaliland. It consists of four components: 1) Adaptation and validation of the sub-Saharan Africa maternal near miss tool for community settings. 2) A longitudinal cohort study of pregnant women. 3) Co-creation and pilot-testing counselling materials aimed at improving the uptake of postpartum contraception. 4) Conducting a secondary analysis triangulating findings from all studies to gain a comprehensive understanding of maternal health in Somaliland informing policy and action. This figure was conducted by ChatGPT (OpenAI), June 2026.

#### Project timeline

The study consists of three sub-studies aligned with specific objectives 1–3, as illustrated in Figure 1. Sub-study one focuses on adaptation of the MNM tool through a Delphi process and validation of the tool through narrative interviews with women and expert evaluation. The adapted MNM components will be embedded within the pregnancy cohort tool (sub-study two).

Sub-study two comprises the pregnancy cohort and begins once the adapted MNM components have been integrated. Data collection will extend over approximately 15 months, including 3 months for recruitment and follow-up until 3 months postpartum, after which data analysis will be conducted.

Sub-study three involves the co-creation process, including iterative cycles of content development, feedback, and pilot testing over an estimated six-month period. This sub-study will draw on findings from the pregnancy cohort to inform the co-creation process. Although the sub-studies overlap in timing, the total study duration is estimated to be 21 months.

### Community-based maternal near miss tool

The MNM framework was developed to understand the underlying reasons for maternal mortality and morbidity [37]. Although adapted for sub-Saharan Africa, its application has largely been limited to facility-based settings [38,39]. This presents a critical evidence gap, as many maternal deaths and complications in low-resource contexts occur outside the formal health system, and complications occurring inside facilities are often poorly recorded [40,41]. Expanding the MNM framework to capture community-level cases could therefore provide a more comprehensive understanding of maternal morbidity and inform context-specific interventions.

Therefore, a community-based MNM identification tool will be developed using a Delphi consensus process [39]. The tool will be based on the sub-Saharan Africa MNM criteria but adapted for use beyond hospital settings [37]. The tool will be integrated into the longitudinal cohort study described below.

Content validity of the community based MNM tool will be established through a Delphi process involving a panel of more than 30 experts in reproductive health from diverse sub-Saharan African contexts. International experts with experience working in low- and middle-income settings will be identified based on relevant publications within the field through PubMed searches and peer recommendations. The process will be coordinated by JK with support from a committee of reproductive health researchers. In round one, clinical symptoms from the existing MNM tool and additional parameters identified through a literature review will be rated by the panel on a 5-point Likert scale. Parameters will be categorized as accepted (median = 5 points), potentially accepted (median = 4 points), or rejected (median ≤ 3 points) and panelists will be able to suggest comments and new parameters. Following, questions will be refined and reorganized by the committee. In round 2, parameters with ≥70% agreement will be included, those with <60% agreement will be excluded, and those with 60–69% agreement will be refined and carried forward to the third round for final yes or no voting. Items that have already reached consensus will be shared for information only. Final decisions will be based on a ≥ 70% agreement threshold for inclusion.

To assess criterion validity, the performance of the adapted community based MNM tool will be evaluated against a reference standard. Within the cohort, women identified by the tool as MNM cases, together with a sample of women classified as non-MNM (screen-negative), will be selected for further assessment. These women will undergo narrative interview to reconstruct care pathways including delays in hours/days and clinical outcomes. And expert review committee will conduct a clinical audit of each case using all available information from the narratives and apply the adapted MNM criteria to determine the ‘true’ MNM status. This expert consensus classification will serve as the reference standard. Cases without consensus will be excluded from the validity analysis. Criterion validity will then be assessed by comparing the MNM classifications from the community-based tool with the expert reviewed reference standard, enabling estimation of diagnostic performance measures, including sensitivity and specificity. This validation component will be embedded within the cohort as a nested criterion validity study.

### Prospective longitudinal cohort study

#### Power calculation

While the study is designed as a prospective cohort with multiple planned sub-analyses, the sample size calculation is based on the primary objective of assessing the association between antenatal care (ANC) utilization and adverse maternal and neonatal outcomes among pregnant women. As individual adverse outcomes such as maternal mortality and MNM are relatively rare, the study will use a composite adverse pregnancy outcome comprising MNM (as defined in specific objective 1), maternal mortality, stillbirth, neonatal death, preterm birth, low birth weight, and neonatal intensive care unit admission.

The sample size is calculated using the double population proportion formula for cohort studies. The calculation assumes an adverse pregnancy outcome proportion of 20% among women attending fewer than four ANC visits and 10% among women attending four or more ANC visits. These assumptions are informed by previous studies from Somaliland and sub-Saharan Africa reporting adverse pregnancy outcome proportions ranging from 10% to 30%, depending on the study population characteristics and outcome definitions [7,42,43]. Assuming a 95% confidence level, 80% statistical power, and a 1:1 ratio between exposure groups, the minimum required sample size is estimated at 438 women. After applying a design effect of 1.5 to account for clustering, the adjusted minimum sample size increased to 657 women. To allow for potential loss to follow-up and incomplete outcome data, the final target recruitment sample is increased to 800 pregnant women.

#### Sampling

This study will be conducted in four of Hargeisa’s eight administrative districts, with two representing urban settings and two representing semi-urban settings. These areas mainly include the settled population. Within each selected district, four sub-districts will be randomly chosen, resulting in a total of 16 of the 31 included sub-districts.

Eligible participants are all pregnant women residing in the selected sub-districts with a gestational age less than 28 weeks at the time of recruitment. Within each of the four selected districts, two community midwives who reside in the selected areas and know about pregnant women will be recruited to assist in identifying pregnant women in their community. The community midwives will list the pregnant women with gestational age assumed to be less than 28 weeks. Throughout data collection, gestational age among included women will be continuously monitored to ensure recruitment of women also during the first trimester. If a woman agrees, her geographical coordinates and phone number will be recorded, and the research team will then visit her home one of the following days with a portable ultrasound scanner to confirm gestational age and thereby whether she is eligible for recruitment. Although first-trimester ultrasound is the gold standard, ultrasound performed up to 28 weeks of gestation still provides sufficiently reliable gestational age estimation, typically ±10–14 days, and is markedly more accurate than menstrual-based dating [44]. Therefore, including women below 28 weeks offered the best balance between feasibility and methodological accuracy. The Crown Rump Length will be used to determine gestational age for women below 12 weeks of gestation, while Biparietal Diameter will be used for women at or after 12 weeks of gestation. If an eligible woman is not present at one of the visits, a revisit will be scheduled. A maximum of three visits will be made.

#### Data collection

**Figure 2.**
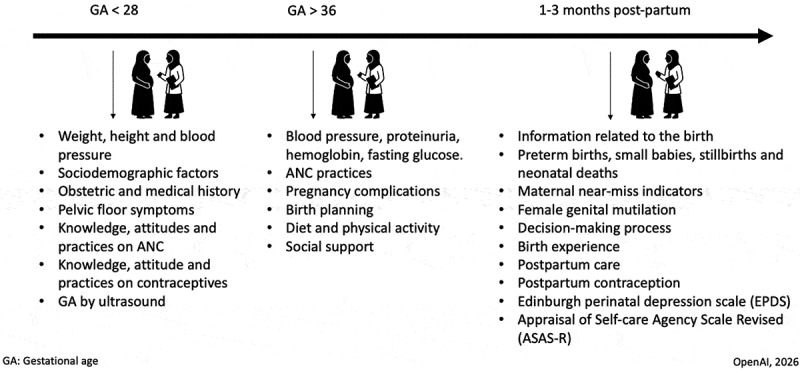
Prospective longitudinal cohort study of pregnant women in Hargeisa, Somaliland. Overview of data to be collected. This figure was conducted by ChatGPT (OpenAI), June 2026.

The cohort includes three visits to each woman’s home, and questionnaires will be developed for each visit through several rounds of adjustments and pilot testing to ensure precision and clarity. All questionnaires will be pilot tested on 60 women in phase one (including ultrasound scans) and 20 women in phase two and three. The questionnaires include sociodemographic factors; questions related to pregnancy, birth, and the postnatal period; experiences and perceptions of FGM; pelvic floor symptoms; and contraception. The questionnaires also include validated Multidimensional Scale of Perceived Social Support (MSPSS), Appraisal of Self-care Agency Scale Revised (ASAS-R), and Edinburgh Perinatal Depression Scale (EPDS). Furthermore, weight, height, blood pressure, blood glucose, and hemoglobin levels will be measured. The full content of the three questionnaires is outlined in Figure 2.

Data collection will be conducted by interviewing women in person. Data collection is divided into three phases. Phase one when women are pregnant below 28 weeks of gestation, phase two when women are in late pregnancy (after 36 weeks of gestation) and phase three when women are between 1 and 3 months postpartum.

A physical examination, including anthropometric measurements (weight and height), blood pressure, and blood and urine analyses (hemoglobin, overnight fasting glucose, and proteinuria) will be carried out during the first and second phases of data collection. If abnormal findings, the research team will refer the women to the nearest health facility or hospital for further management.

Each pregnant woman will be provided with a personal ID to enable follow-up during the different phases of data collection. A tracking sheet will be developed in Excel to ensure follow-up of each woman. As majority of women in Hargeisa have access to a phone, women will be phoned to schedule follow-up visits for the second and third interviews. If the woman is not reachable by phone, the research team will visit her using GPS coordinates (KoboToolbox).

Ultrasound scans measuring Biparietal Diameter will be anonymized and reviewed by VR and AWL using the Butterfly platform. In cases where image quality is suboptimal, the images will be reviewed in detail during Zoom meetings with participation from FM, VR, AWL, and the research assistants. Recommendations will be made based on consensus.

The questionnaires will be filled out on paper by the research assistants while interviewing the women. All questionnaires will be checked by MM and FM, and if there are missing data, the team will return to the pregnant woman. FM will supervise data collection and will be in the field during all phases of data collection. Data will be entered in REDCap by a trained research assistant and subsequently verified by an independent research assistant through thorough review of all hard-copy questionnaires and corresponding entries. Additional data validation will be conducted using frequency tabulations to identify missing values and outliers. Identified discrepancies will be corrected by rechecking the hard-copy questionnaires and by performing cross-tabulations of interrelated variables. Monthly meetings will be held among the research team to assess inclusion and follow-up. Additionally, FM and the research assistants will meet weekly.

#### Variables

Sociodemographic variables include educational level, occupation and monthly income of both the woman and her husband as well as housing conditions and household assets.

Whenever possible, the National Guidelines on ANC of Somaliland will be used as reference [45]. Adequate ANC is defined as ≥4 ANC visits, and timing of ANC visits will be divided into first visit at ≤12 weeks’ gestation versus >12 weeks’ gestation. Pregnancy complications include diabetes (pre-gestational and gestational) and hypertensive disorders of pregnancy (HDP) (essential hypertension, gestational hypertension, pre-eclampsia, and eclampsia). Gestational diabetes is defined as fasting blood glucose level >92 mg/dL (5.1 mmol/L). HDP includes all women with a blood pressure >90 diastolic or >140 systolic [45]. Pre-eclampsia is defined as women with a blood pressure >90 diastolic or >140 systolic cooccurring with proteinuria [45]. ‘Pre-eclampsia with severe features’ is defined as pre-eclampsia with a description of at least one of the following symptoms: severe headache, swelling of the face, upper abdominal pain or oliguria (less that a table spoon) [45]. MNM indicators will be defined using a Delphi study (see previous section).

Birth outcomes include admission to neonatal intensive care unit, mode of birth (vaginal birth, assisted vaginal birth, and cesarean section), postpartum hemorrhage, eclampsia, and maternal death. Stillbirth, neonatal death within 1 and 7 days, preterm birth < week 37, and low birth weight <2000 grams will be assessed either as complications or as adverse birth outcomes, depending on the analysis.

Postpartum depression will be assessed using EPDS [46]. Social support will be assessed using the MSPSS tool, which contains 12 items rated on a 7-point Likert scale (1 = very strongly disagree, 7 = very strongly agree) [47]. Based on the mean item scores, the following categories will be used: low support (<3), moderate support (3–5) and high support (>5). Self-care will be assessed using the ASAS-R score, which is a 15-item scale that appraises the enabling traits of self-care agency, and uses a Likert scale ranging from 1 (strongly disagree) to 5 (strongly agree), with total scores ranging from 15 to 75, with higher scores indicating greater self-care agency [48].

Collective decision making distinguishes between decisions made by the women and other people. Each decision-maker category (e.g. the woman herself, husband/partner, parents, in-laws, grandmother, friends) will be coded as 1 = involved and 0 = not involved and will be summed to create a variable representing the total number of people involved. The variable will initially be treated as continuous to assess potential relationships between increasing involvement and the likelihood of SMOs. If the distribution is skewed or non-linear, categories will be created (e.g. low involvement (1–2 people) vs. high involvement (≥3 people)).

The adapted MNM tool components will be integrated into the cohort-questionnaires to identify potential MNM cases in the pregnancy cohort and enable validation of the tool.

### Co-creating contraceptive counselling materials

We will use the PartoMa iterative co-creation framework and the PRODUCES+ Evidence-based Co-Creation for Public Health Guideline to apply the cohort findings for co-creating postpartum contraceptive counselling materials and pilot test their feasibility and effects [49,50]. As previous evidence from Somaliland and similar contexts has identified substantial unmet need for postpartum family planning, likely driven by restricted reproductive agency among women, we find this intervention focus to be relevant for this initial pilot testing of the potential, strengths and challenges in co-creation within reproductive healthcare in Somaliland.

Existing counselling materials in Somaliland, scientific literature on counselling strategies for postpartum contraception and findings from the PROMISE cohort regarding women’s reproductive experiences, decision-making processes, psychosocial circumstances, and barriers to care will directly inform the initial draft, which will be prepared by the research team. Iterative co-creation workshops and focus group discussions will follow, with co-creators engaged either through home visits or by participating in workshops emphasizing joint decision-making and equal contribution. Co-creators will include community representatives (women and men), healthcare providers, policymakers, religious leaders, non-governmental organizations, and international academic researchers.

Member checking (i.e. checking back with the co-creators to make sure that the data, insights, or design outputs truly reflect their perspectives) and face validity assessments (i.e. whether the outcomes, tools, or processes developed seem credible and appropriate to the stakeholders involved) will be conducted at each stage, and WhatsApp groups will facilitate ongoing feedback. The materials will be revised until no major concerns remain and then submitted for approval by the Ministry of Health in Somaliland.

The quality of the co-creation process will be evaluated through semi-structured interviews exploring interactions, decision-making, perceived inclusiveness, and the extent to which outputs reflect shared goals.

The finalized materials will be piloted among 10–15 women to assess feasibility, comprehension, and acceptability. A small-scale effectiveness evaluation will then compare women who receive counselling with those who do not receive counselling. Pilot testing will be conducted during routine postpartum healthcare visits postpartum. Outcomes will include contraceptive wishes, plans, and use as well as knowledge of methods, with barriers explored through in-depth interviews. Sample size calculations are based on the outcome ‘wish to use contraceptives,’ assuming an increase from 15% to 30% with the intervention, requiring 118 women per group (reaching a power of 80% and alpha of 5%). These assumptions will be refined using PROMISE cohort data, and primary outcomes may be adjusted based on co-creation findings. Successful piloting will inform the design of future large-scale evaluations and further co-creation of reproductive health interventions in the Somaliland context.

### Secondary analysis of the PROMISE studies

Based on the results of specific objectives 1–3 challenges and opportunities will be explored with the aim of developing recommendations that can be used to inform policy and action plans for reproductive health in Somaliland.

### Analyses

Quantitative data from the cohort will be cleaned, anonymized, and analyzed using statistical software, such as SPSS or STATA. Descriptive analyses will be carried out. The Pearson chi-square test will be applied for categorical variables and the independent t-test for continuous variables to determine statistically significant differences between the groups. Pearson chi-squared test and Bi- and multivariate logistic regression will examine associations between exposures and outcomes. Confounders will be identified according to the association being assessed. A *p*-value of <.05 will be considered statistically significant.

The sensitivity, specificity, and predictive values of the MNM tool will be calculated by comparing findings from the tool with narrative interviews. The number of individual SMOs will be analyzed using descriptive statistics, and associations between SMOs and collective decision-making will be analyzed using logistic regression analysis.

To facilitate the translation of findings, a secondary analysis triangulating findings from all sub-studies will be conducted and recommendations generated.

### Policy and action

In addition to publications in peer-reviewed, open-access journals, dissemination seminars will be held in Somaliland. The study’s main findings will be shared with local, national, and international stakeholders, including frontline health providers, selected community authorities, research participants, and the wider community.

## Discussion

This study addresses major evidence gaps in reproductive health in Somaliland, where maternal and perinatal mortality remain unacceptably high, and coverage of key services such as ANC, facility-based birth, and postpartum contraception remains critically low. The protocol outlines an integrated longitudinal design that explores reproductive health challenges, including pregnancy, childbirth, postpartum health, contraceptive use, FGM, and pelvic floor symptoms. The study situates these issues within the broader social context, examining the roles of social support, self-care, and collective decision-making. These factors are important in the context of Somaliland and within which maternal health must be understood.

By triangulating individual, sociocultural, and structural factors, this study will generate comprehensive in-depth analyses. The use of a shared pregnancy cohort across multiple research objectives further enables generation of rich, integrated data. Rigorous quality assurance processes, including ultrasound verification of gestational age, external review of scans, double entry of data, continuous monitoring, and close field supervision, further enhance the validity and reliability of the data.

Another strength is the ambitious generation of community-based data in a setting without population registers. For example, the prevalence of gestational diabetes and HDP in Somaliland is unknown and community studies are essential to capture the true burden of disease. Bridging knowledge gaps is a crucial step toward mobilizing political will to strengthen maternal and perinatal health services.

The study also entails several limitations. First, recruitment is restricted to Hargeisa, which may not capture the diversity of experiences across Somaliland’s rural and nomadic populations. Second, loss to follow-up is a risk, particularly during the postpartum period, despite strategies to maintain contact. The 2-month time interval for the postpartum interview, chosen for pragmatic reasons, makes it difficult to compare women within the cohort. Data are largely self-reported, introducing potential recall or social desirability bias. The use of local enumerators may inadvertently contribute to selection bias during recruitment. Since Somaliland has strong community-networks, we believe that the vast majority of women will be identified, while knocking on all doors was deemed too time-consuming. Logistical challenges, such as reaching women during late pregnancy before delivery, may affect the collection of key measurements (e.g. hemoglobin, blood pressure, and blood glucose). Participant fatigue and the health status of women may also limit data completeness, although efforts will be made to adapt the interviews to women’s preferences and well-being.

Several questions are sensitive which may affect the reliability of the data. To mitigate this, interviews will be conducted in private settings, confidentiality will be emphasized, and participants will be given the option to skip questions. The co-creation of postpartum contraceptive counselling materials may encounter resistance from stakeholders, including religious leaders. To address this, the co-creation process will be designed to foster respectful dialogue and mutual understanding, aiming for counselling materials acceptable, feasible, and sustainable within the Somaliland context. Co-creation further strengthens translation of evidence into practice by increasing community and stakeholder ownership. Even if compromises are required, this process will generate important insights into contraceptive knowledge gaps and reproductive agency.

Growing evidence from global health implementation science demonstrates that interventions co-designed with communities and frontline implementers are more acceptable, context-adapted, and sustainable [51]. Co-creation processes can shift the power within knowledge generation, ensure cultural alignment, and increase the likelihood of real-world uptake [52]. In the Somaliland context, where reproductive decisions are collectively shaped and influenced by gender norms, religion, and clan structures, co-creation is particularly important to ensure that interventions are trusted, feasible, and actionable.

Finally, the MNM tool will be adapted through a Delphi process, drawing on the expertise of researchers previously involved in adapting the tool for sub-Saharan Africa. As the MNM framework was originally developed for clinical settings, there is a risk that women may not provide sufficient information for an accurate case classification. Nevertheless, if the adapted tool demonstrates acceptable validity, this study will represent the first attempt to systematically capture MNM at the community level with important implications for future practice.

## Conclusion

The PROMISE study will generate contextually grounded community-based evidence on maternal and reproductive health in Somaliland, addressing critical gaps in knowledge regarding pregnancy complications, contraceptive use, and psychosocial determinants of care. Through a longitudinal cohort design and co-created interventions, the project aims to inform context-specific and sustainable strategies to strengthen reproductive health and agency in Somaliland and beyond.

## Data Availability

Data sharing not applicable – no new data generated.
